# Relative bench-press strength and jump power are associated with distinct tracking-based in-game shares in professional ice hockey

**DOI:** 10.3389/fspor.2026.1917193

**Published:** 2026-10-13

**Authors:** Peter Spornberger, Hendrik Kolbert, Thimo Wiewelhove

**Affiliations:** 1 Department of Fitness and Health, IST University of Applied Sciences, Düsseldorf, Germany; 2 Medical and Athletic Department, Schwenninger Wild Wings, Villingen-Schwenningen, Germany

**Keywords:** expected goals, Fenwick, ice hockey, local positioning system (LPS), off-ice testing, performance analysis, player tracking, team sport analysis

## Abstract

**Background:**

Ice hockey is an intermittent, high-intensity team sport, yet the transfer from preseason off-ice testing to tracking-derived in-game impact metrics remains underexplored in professional settings.

**Objective:**

To examine associations between preseason off-ice fitness and tracking-derived 5-on-5 in-game impact in a Deutsche Eishockey Liga (DEL) team across three seasons.

**Methods:**

Retrospective analysis of 28 male professional skaters (*N* = 50 player-seasons after listwise deletion, up to three observations per player). Predictors were 30 m sprint, countermovement jump (CMJ), relative single-leg strength (Bulgarian split squat 1RM per body mass, SLS/BM), relative upper-body strength (bench press 1RM per body mass, BP/BM), estimated maximal oxygen uptake (VO₂max), body fat percentage, and age. Outcomes were two team-relative share metrics derived from the league's official player-tracking system: the relative expected-goals-for percentage (xGF%Rel), defined as a player's on-ice share of expected goals minus the team's share with the player off the ice, capturing chance quality; and the relative Fenwick-for percentage (FF%Rel), the corresponding on-minus-off share of unblocked shot attempts, capturing territorial play. Ordinary least squares models included season fixed effects and player-clustered robust standard errors; multiplicity across predictors was controlled using the Holm procedure. Sensitivity models additionally adjusted for playing position.

**Results:**

Model fit was modest (adjusted R^2^: 0.16 for xGF%Rel; 0.21 for FF%Rel). After Holm correction, BP/BM was positively associated with xGF%Rel (*β* = .021, p_Holm = .011). For FF%Rel, CMJ was positively associated (*β* = .018, p_Holm = .012) and estimated VO₂max negatively associated (*β* = −.019, p_Holm = .035). Sensitivity analyses preserved the direction and approximate magnitude of all three associations. Statistical support was uniform only for CMJ–FF%Rel, which remained significant throughout; the BP/BM association did not survive allometric scaling and the estimated-VO₂max association fell just above the adjusted threshold after author exclusion.

**Conclusions:**

In this single-team DEL cohort, preseason off-ice fitness showed metric-specific rather than global associations with 5-on-5 relative share outcomes. Relative upper-body strength aligned with chance-quality share, while lower-limb explosiveness aligned with territorial share; the inverse VO₂max association likely reflects role and deployment context rather than detrimental effects of aerobic capacity *per se*. Findings are associative, not causal, and warrant validation in larger multi-team datasets with explicit usage/context adjustment.

## 1 Introduction

Ice hockey is an intermittent, high-intensity team sport characterized by repeated bouts of explosive effort interspersed with brief recoveries (1). Typical shifts last ∼30–80 s, with cumulative on-ice exposure of ∼15–25 min per game, placing substantial demands on both anaerobic and aerobic energy systems (2–4). Accordingly, conventional off-ice assessment batteries prioritize lower-extremity power, linear sprint performance, maximal force production, and aerobic capacity—domains that are systematically employed in talent identification and high-performance athlete profiling frameworks (5, 6). Nevertheless, empirical links between off-ice measures and on-ice outcomes remain inconsistent: jump and sprint indices often correlate with linear skating speed, but effect sizes and external validity vary across cohorts, age groups, and protocols (7–13). Moreover, aerobic capacity supports recovery and repeated-shift ability, and evidence syntheses indicate a modest, consistent translation from off-ice aerobic assessments to on-ice endurance proxies (4, 7, 14). Although specific off-ice metrics can show modest transfer to corresponding on-ice parameters, a broader challenge lies in explaining the full spectrum of fitness–in-game performance relationships.

A 17-study scoping review found only modest transfer from off-/on-ice tests to selection and in-game performance and explicitly argues for context-linked indicators (14). Consistent with this, prior work in Norwegian men's professional hockey indicates that generic off-ice tests exhibit at best trivial-to-small associations with points per game (P/GP), while age is the dominant correlate; although national-team-selected players display higher strength, fitness adds little predictive value beyond age (15). Similarly, in a cohort of 21 elite junior males, preseason off-ice fitness showed little direct association with in-season game metrics, whereas on-ice skating speed correlated with multiple outcomes: faster forward speed related to higher expected goals (and more received hits), and faster backward speed among defensemen aligned with greater offensive contribution and blocked shots—underscoring the value of on-ice tests alongside off-ice assessments (8).

Concurrently, ice hockey performance analysis has moved beyond box scores (e.g., goals, points, +/-) to tracking-derived, advanced metrics (e.g., Corsi, Fenwick, xGF) that capture territorial dominance, chance quality, and individual performance facets (8, 16). Advanced, team-derived metrics have been applied only sparingly to in-game performance research; one instance is LaPrade and colleagues (17) on NHL return-to-play after hip arthroscopy. Despite extensive work on the transfer of off-ice fitness to on-ice skating performance, evidence linking standard preseason off-ice test batteries to tracking-derived, advanced 5-on-5 impact metrics in professional ice hockey remains scarce. The most closely related study addressed junior players using notational (video-tagged) game data and bivariate correlations, and related predominantly on-ice skating tests—rather than off-ice fitness—to on-ice expected-goals and shot-based indicators (8). This paucity may reflect the historical unavailability of fine-grained tracking-based match data (1). For instance, league-wide deployment of player-tracking infrastructure in European professional competitions is a comparatively recent development, with the German Deutsche Eishockey Liga (DEL) implementing such systems across the league beginning in 2022 (18). To address this gap, regression models that link standard preseason off-ice tests to existing tracking-derived advanced metrics were estimated, as these metrics provide more proximal indicators of individual in-game performance than traditional box-score outcomes (16). Building on this approach, commonly used preseason off-ice tests were linked to tracking-derived 5-on-5 impact, operationalized via team-relative share metrics. Specifically, relative expected goals for percentage (xGF%Rel) as a measure of chance-quality impact and relative Fenwick for percentage (FF%Rel) as a measure of unblocked shot-attempt share (territorial play) were examined. The aim of this study was therefore to determine whether, and which, preseason off-ice fitness measures are associated with tracking-derived 5-on-5 impact metrics in a professional DEL team across three seasons. The study was designed as a retrospective, exploratory, hypothesis-generating association study; it is neither a validation study of the testing battery or the tracking system, nor does it permit causal inference about training effects. Its intended practical value is to inform strength and conditioning coaches and sporting management about which routinely collected off-ice tests carry explanatory value for in-game impact — and which do not — thereby providing an evidence-informed basis for streamlining preseason testing batteries and prioritizing monitoring resources. The primary hypothesis is directional: better preseason off-ice fitness is associated with more favorable 5-on-5 impact. Specifically, faster 30 m sprint performance, greater lower-limb power (countermovement jump, CMJ), and greater relative strength (Bulgarian split squat/body mass, SLS/BM and bench press/body mass, BP/BM) are expected to relate positively to xGF%Rel and FF%Rel, whereas higher body fat percentage is expected to relate negatively. Estimated VO₂max is included as a proxy for aerobic capacity, and age as a non-modifiable career-stage predictor. These prespecified associations were tested using regression models with season fixed effects and player-level dependence accounted for. The predictors, directional hypothesis, and analysis plan were defined *a priori* (though the study was not preregistered), so inference remains exploratory and hypothesis-generating rather than confirmatory.

## 2 Materials and methods

### 2.1 Sample

Twenty-eight male professional ice hockey players from a club in the German Deutsche Eishockey Liga (DEL) were included in the analysis. The cohort comprised 17 forwards and 11 defensemen, with no goaltenders (Table 1). All players were full-time professionals for whom ice hockey was their primary occupation, competing in the DEL, Germany's top-tier professional men's league, which is ranked among the top five European leagues (19)). The Participant Classification Framework by McKay and colleagues (20) classifies team-sport athletes primarily by team representation and level of competition. Accordingly, each participant was classified by the highest applicable criterion: Every player met at least one Tier 4 (Elite/International Level) criterion: competition in an international league or tournament (Champions Hockey League), competition at the international level for a national team, or prior NCAA Division I competition. Domestic DEL competition, which corresponds to the national-league (Tier 3) criterion, provided the common professional context but was not the criterion establishing Tier 4. On this basis, and following the framework's guidance to classify a cohort by its typical member and to select the higher tier where participants span adjacent tiers, the sample was classified as Tier 4: Elite/International Level (20). The observation period comprised three consecutive seasons: 2022/23, 2023/24, and 2024/25. Over this interval, the team can be characterized as a mid-table, average-performing team, with final league positions ranging from 12th to 6th (21). No *a priori* sample-size or power calculation was performed; the study size was determined by data availability and comprised all player-seasons from the participating club that met the inclusion criteria across the three observed seasons (a census of the eligible cohort rather than a sampled subset). Only skaters (forwards and defensemen) were included; goaltenders were excluded because their physical demands, on-ice roles, and tracking-based performance metrics differ fundamentally from those of skaters. Participants were eligible for inclusion if they completed the preseason off-ice fitness testing and subsequently remained on the club's roster for one season with participation in ≥50% of regular-season games (≥28 of 56 games in 2022/23; ≥26 of 52 games in 2023/24 and 2024/25). Differences in season length reflect COVID-19–related adjustments to relegation rules in 2021/22, which resulted in 15 rather than the usual 14 teams competing in the league during the 2022/23 season (21–23). Across the three observed seasons, 43 players were rostered by the club (76 player-seasons). Fifteen players (22 player-seasons) did not meet the inclusion criteria — participation in <50% of regular-season games and/or non-completion of preseason off-ice fitness testing — leaving 28 players (54 player-seasons). A further four player-seasons from three players were excluded by listwise deletion owing to incomplete off-ice fitness test data (≥1 test not completed), yielding a final analytic sample of *N* = 50 player-seasons (Supplementary Material). Six players contributed data across three seasons, ten across two seasons, and twelve in a single season. Off-ice fitness data were provided to the research team by the club's head strength and conditioning coach as an electronic dataset compiled from the club's routine preseason testing records; in-game, player-level performance data were provided via the club by the league's official vendor, Wisehockey (24). Both the club and Wisehockey granted written permission to use the data for research purposes. The study used de-identified retrospective records and was classified as a non-interventional secondary analysis (see Ethics statement).

**Table 1:** Sample description (after listwise deletion, *N* = 50).

Variable	Overall Mean (SD)	Overall 95% CI	Defensemen Mean (SD)	Defensemen 95% CI	Forwards Mean (SD)	Forwards 95% CI
Age [years]	28.44 (4.46)	[27.17; 29.71]	30.00 (4.68)	[27.74; 32.26]	27.48 (4.11)	[25.98; 28.99]
Height [cm]	184.58 (5.48)	[183.02; 186.14]	186.53 (4.79)	[184.22; 188.83]	183.39 (5.61)	[181.33; 185.44]
BM [kg]	86.60 (7.85)	[84.37; 88.83]	90.83 (5.28)	[88.28; 93.37]	84.00 (8.10)	[81.03; 86.97]
BMI [kg/m^2^]	25.38 (1.56)	[24.94; 25.83]	26.10 (0.99)	[25.62; 26.58]	24.95 (1.70)	[24.32; 25.57]
Body fat [%]	9.99 (2.77)	[9.20; 10.77]	10.86 (2.78)	[9.52; 12.20]	9.45 (2.67)	[8.47; 10.43]

The sample comprised 50 overall player-seasons, including 19 defensemen-seasons and 31 forwards-seasons. Because players could contribute data from multiple seasons, the 50 overall player-seasons were derived from 28 unique players. BM, body mass; BMI, body mass index; CI, confidence interval; SD, standard deviation.

### 2.2 Study design

A retrospective, longitudinal observational study was conducted. This study is reported in accordance with the Strengthening the Reporting of Observational Studies in Epidemiology (STROBE) guidelines for cohort studies; the completed checklist and a flow diagram of player-season selection are provided as Supplementary Material. The DEL competitive year follows an annual cycle of preseason (preparation, late July to mid-September), regular season (mid-September to early March), play-offs for qualifying teams (early March to late April), and an off-season recovery phase. Off-ice testing was conducted once per season, at the beginning of the preseason (typically late July to early August), before the resumption of on-ice team training, whereas all in-game metrics were derived exclusively from regular-season games and aggregated into player-season summaries. The testing location, measurement instruments, supervising instructor, testing sequence, and timing were kept constant across all observed seasons.

### 2.3 Procedures

#### 2.3.1 Off-Ice testing

Testing was conducted on two consecutive days from 9:00 to 13:00 under the supervision of the club's strength and conditioning coach (≥10 years experience with the team). Players were instructed to maintain their usual sleep, hydration, and nutrition routines; no off-ice or on-ice practice was performed on the day preceding testing or during the two testing days. Prior to each test, players completed their habitual individual warm-up. In-sample reliability was not quantified. Test administration nonetheless followed standardized procedures (fixed test order, rest intervals, verbal instructions, and predefined technique/range-of-motion criteria), and the battery comprised protocols commonly used in ice-hockey and team-sport off-ice testing, for which acceptable reliability has generally been reported under standardized conditions (25). Familiarization trials were not conducted because players had prior experience with the testing battery. However, typical error and test–retest reliability were not established for the present instruments and cohort, and no reliability estimates were available for the single-leg (Bulgarian split squat) strength test or the cycle-ergometer VO₂max estimate. The absence of in-sample reliability is therefore treated as a limitation, and near-threshold effects are interpreted against potential measurement error accordingly (see Section 4.1).

On Day 1, body mass (kg) and standing height (cm) were measured, and body fat percentage was estimated using the Jackson-Pollock seven-site skinfold method (26) with a calibrated caliper; body mass index (BMI) was calculated as kg/m^2^. Jump and sprint testing followed: a broad jump (BJ; standing long jump to a stable landing), squat jump (SJ), and CMJ were performed with hands on hips, with jump height recorded on a contact mat (Jump Mat Pro, FSL Scoreboards Ltd., Cookstown, Northern Ireland, UK). For the SJ, players initiated from a static ∼90° knee angle; for both SJ and CMJ, landings were executed with extended knees as instructed. Linear sprints were recorded using electronic timing gates (Witty Gate, Microgate Srl, Bolzano, Italy) at 0, 10 m, and 30 m, with a self-initiated start 0.5 m behind the first gate and split times recorded at 10 m and 30 m. Three attempts were performed for BJ, SJ, and CMJ with 15-s rest between jumps, and two attempts for the 30 m sprints with 3-min rest between trials; the best performance was retained.

Strength testing followed: The SLS and BP were assessed with a 5RM protocol after self-selected warm-up and approach sets. Repetitions were required to be technically valid (full control, full ROM, no assistance); unsuccessful attempts were repeated after rest. For SLS, participants performed a Bulgarian split squat with a self-selected stance distance from the rear-foot support, and the knee of the supporting leg was required to contact a floor-mounted pad on every repetition. Both legs were tested; if values differed, the mean of the two trials was used. For BP, grip width was self-selected, and range of motion was standardized by requiring the bar to contact the chest on each repetition. Although grip width (BP) and stance distance (SLS) were self-selected to reflect athletes’ habitual technique, they were kept consistent within each player across seasons where possible and recorded by the staff for standardization. One-repetition maximum (1RM) was estimated deliberately with exercise-matched equations: the bench-press-specific Mayhew equation (27) for BP, and the general Brzycki equation (28) for SLS, as no repetition-based 1RM equation validated for the unilateral Bulgarian split squat exists; its use there constitutes an extrapolation. Relative strength was expressed as SLS/BM and BP/BM (1RM divided by body mass). Because both lifts were tested at 5 repetitions, repetition-based prediction error was minimized (29); the equation-specification error for SLS is nonetheless unquantified and plausibly larger, so SLS/BM values were interpreted with additional caution. Pull-ups were performed with a pronated grip (self-selected width) for maximal body-weight repetitions to failure.

On Day 2, a cycling test was administered on a cycle ergometer (M3i, Keiser, Fresno, CA, USA) to obtain an estimated VO₂max value. After a 5-min warm-up at 100 W, a staged protocol commenced at 150 W with 50-W increments every three minutes to volitional exhaustion or inability to sustain the target power. Cadence was targeted at ∼90 rpm; standardized verbal encouragement was provided. Heart rate was monitored with a chest-strap sensor (Firstbeat Technologies Oy, Jyväskylä, Finland), and an estimated VO₂max value (mL·kg⁻^1^·min⁻^1^) was obtained from heart rate, body mass, and achieved cycle-ergometer power output using the “Maximal Fitness Test for Cycling” algorithm implemented in Firstbeat Sports [version 4.7.2.1 (3.3.8.3); Firstbeat Technologies Ltd., Jyväskylä, Finland]. The underlying estimation approach is proprietary; the available Firstbeat technical documentation describes VO₂max estimation from heart-rate, body mass and speed data (30), and no validation evidence specific to this cycle-ergometer application was available to the authors. The resulting value is therefore treated throughout as an estimated aerobic-capacity proxy whose validity for this specific protocol has not been established.

Only seven key off-ice/anthropometric predictors were retained to represent distinct physical attributes: Speed (30 m), Explosiveness (CMJ), Lower body strength (SLS/BM), Upper body strength (BP/BM), aerobic capacity (estimated VO₂max), body composition (body fat percentage) and age. Other collected tests (e.g., BJ, pull-ups) were not included to reduce redundancy with CMJ/strength measures and to preserve power and model stability in a small clustered sample (Table 2).

**Table 2:** Descriptive statistics (after listwise deletion, *N* = 50).

Variable	M	SD	95% CI
Age (y)	28.440	4.459	27.173; 29.707
Height (cm)	184.580	5.481	183.022; 186.138
BM (kg)	86.596	7.847	84.366; 88.826
BMI (kg/m^2^)	25.384	1.563	24.939; 25.828
Body fat (%)	9.986	2.769	9.199; 10.773
BJ (cm)	263.960	13.754	260.051; 267.869
SJ (cm)	46.636	5.622	45.038; 48.234
CMJ (cm)	48.688	6.223	46.919; 50.457
10 m (s)	1.837	0.079	1.815; 1.860
30 m (s)	4.273	0.143	4.233; 4.314
SLS/BM (rel)	1.516	0.201	1.458; 1.573
BP/BM (rel)	1.287	0.150	1.244; 1.330
Pull-ups (Repetitions)	17.220	5.452	15.671; 18.769
Estimated VO₂max (mL·kg⁻^1^·min⁻^1^)	57.448	6.470	55.609; 59.287
xGF%Rel	−0.0061	0.0541	−0.0215; 0.0093
FF%Rel	−0.0045	0.0371	−0.0150; 0.0060

BJ, broad jump; BM, body mass; BMI, body mass index; body fat %, body fat percentage; BP/BM, bench press/body mass; CI, confidence interval; CMJ, countermovement jump; FF%Rel, relative Fenwick for percentage; M, mean; SD, standard deviation; SJ, squat jump; SLS/BM, single-leg squat/body mass; VO₂max, maximal oxygen uptake; xGF%Rel, relative expected goals for percentage.

#### 2.3.2 In-game performance metrics

In-game data were provided by Wisehockey [Wisehockey Oy, Tampere, Finland (24);] using player-worn and puck-worn tags in combination with a system of 20–28 in-arena Quuppa locators (Quuppa Oy, Espoo, Finland) to derive spatiotemporal event data from official DEL games starting in the 2022/23 season (18). Player-season variables used in this study comprised xGF%Rel and FF%Rel. To clarify the respective contributions: all in-game metrics used in this study, including the relative share outcomes (xGF%Rel, FF%Rel), were obtained directly as standard outputs of the Wisehockey system, based on its proprietary event detection and shot-quality (xG) model. No new tracking metrics or models were developed by the author; the study's contribution lies in linking these established, vendor-provided metrics to preseason off-ice fitness data. The formal definitions below are reported for transparency and reflect Wisehockey's computation, not the author's own derivation. Both metrics were computed exclusively at 5-on-5 to avoid confounding effects arising from player deployment on the power play or penalty kill (31, 32). Individual player-level 5-on-5 time-on-ice (TOI) was not part of the standard vendor output available for this study; consequently, per-player 5-on-5 exposure could not be quantified, and player-seasons could not be weighted by 5-on-5 TOI. Because both tracking event detection and xG are proprietary vendor algorithms, version changes across seasons could not be independently audited.

##### 2.3.2.1 xGF%Rel

A skater's on-ice share of expected goals for relative to the team's share when he is off the ice (xGF%Relₚ,ₛ = xGF%ᵒⁿₚ,ₛ−xGF%ᵒᶠᶠₜ,₋ₚ,ₛ; p, player; s, season; t, team); expected goals are computed using Wisehockey's shot-quality model that incorporates shot distance and angle together with pre-/in-shot contextual features (e.g., passes, lateral puck/goalie movement, screens, and goalkeeper positioning). Positive values indicate a higher proportion of expected goals when the player is deployed (33–35).

$$xGF{\text{\%}}_{\;p,s}^{on}=100\cdot \frac{xGF_{\;p,s}}{xGF_{\;p,s}+xGA_{\;p,s}}$$

##### 2.3.2.2 FF%Rel

A skater's on-ice share of unblocked shot attempts—shots on goal and missed shots—relative to the team's share when he is off the ice (FF%Relₚ,ₛ = FF%ᵒⁿₚ,ₛ−FF%ᵒᶠᶠₜ,₋ₚ,ₛ; p, player; s, season; t, team). Because Fenwick excludes blocked shots, FF%Rel is less directly affected by blocked-shot events than Corsi and may be useful for approximating individual involvement in creating shot attempts (33).

$$FF{\text{\%}}_{\;p,s}^{on}=100\cdot \frac{FF_{\;p,s}^{\;for}}{FF_{\;p,s}^{\;for}+FF_{\;p,s}^{against}}$$

Although xGF% and FF% are often expressed on a 0–100 scale, the relative-share outcomes (xGF%Rel & FF%Rel) were retained on a 0–1 proportion scale. Therefore, effects are interpreted in percentage points after multiplication by 100.

### 2.4 Statistical analysis

Data were collected from a single professional ice hockey team across three seasons. Rows with missing values on the outcome or any predictor were excluded (complete-case analysis; listwise deletion), leaving *N* = 50 observations for each outcome from 28 unique players (up to three observations per player). A missingness check compared retained and excluded player-seasons on age, position, season, both outcomes, and each predictor. Because exclusions arose solely from incomplete off-ice testing, both outcomes were observed for all four excluded player-seasons. The excluded player-seasons had higher mean outcome shares than the retained sample, so a bias arising from complete-case analysis cannot be excluded; given only four excluded rows, and a difference driven largely by a single player, the direction and magnitude of any such bias remain uncertain. Complete-case analysis was retained as a transparent primary strategy, mean imputation was not applied, and the potential for residual bias is acknowledged as a limitation. To place predictors on a common scale and reduce leverage effects, all continuous predictors were z-standardized within each analytic sample (mean = 0, SD = 1). Because all continuous predictors were z-standardized, each regression coefficient represents a standardized effect size — the change in the outcome (in proportion units; ×100 for percentage points) associated with a 1-SD increase in the predictor. Speed, e.g., 30 m was inverted so that higher values denote superior capacity (i.e., ‘higher = better’). Descriptive statistics and the primary cluster-robust regression models were computed in jamovi (version 2.6; RRID:SCR_016142) (36), which uses R as its statistical engine. Because the fwildclusterboot package required for the wild cluster restricted bootstrap is no longer available on CRAN and could not be installed into the jamovi R library, the cluster-robust models were additionally reproduced and the wild cluster restricted bootstrap was computed in a separate R session (R version 4.6.1) (37). Cluster-robust variance estimation used the estimatr package (version 1.0.6; function lm_robust) with cluster-robust standard errors of the CR1 (Stata-type) form (se_type = “stata”) and player identity as the clustering variable. Inference for all analytic cluster-robust models used a t distribution with degrees of freedom equal to the number of player clusters minus one [t(G−1)]; the primary and position-adjusted models used t(27) with G = 28, and the author-exclusion models used t(26) with G = 27. Ninety-five-percent confidence intervals were computed as *β* ± t(G−1, 0.975) × SE. The wild cluster restricted bootstrap was implemented with the fwildclusterboot package (version 0.14.3; function boottest), installed from the R-universe developer repository because the package is no longer available on CRAN; the null hypothesis was imposed (restricted bootstrap) with Rademacher weights, resampling at the player-cluster level over 9,999 replications and two-sided *p*-values and 95% confidence intervals were obtained by test inversion. Because the wild bootstrap generates its own reference distribution, the t(G−1) rule does not apply to the bootstrap *p*-values or their test-inverted intervals; these bootstrap results are unadjusted for multiplicity and pertain only to the three selected coefficients. The allometric scaling exponent was estimated by ordinary least squares log–log regression of bench-press 1RM on body mass using base R (lm), with the standard error additionally clustered by player. Multiplicity across the seven predictors within each outcome was controlled with the Holm procedure (p.adjust, method = “holm”). Seasons were modeled as fixed effects (indicator variables). Because players appear in multiple seasons, ordinary least squares (OLS) with cluster-robust standard errors were estimated at the player level (two-sided *α* = .05). Given the player-season structure and the absence of player fixed effects, the regression coefficients are interpreted primarily as between-player associations. Because player-level 5-on-5 TOI was not available in the vendor data, player-seasons could not be weighted by 5-on-5 exposure; each qualifying player-season therefore entered the models with equal weight. While some players are observed across multiple seasons, the models do not isolate within-player changes over time (e.g., training effects) and therefore do not identify causal effects. Variance inflation factors (VIFs) were computed on the standardized predictors to assess multicollinearity. In addition, the familywise error rate was controlled across the seven predictors per outcome using the Holm correction (38). Multiplicity was controlled within, but not across, the two outcomes; the familywise error rate was not adjusted across the full set of 14 predictor×outcome tests. As the outcomes are conceptually distinct yet correlated share metrics, per-outcome correction was adopted; the study-wide false-positive rate is thus not strictly controlled.

As a sensitivity analysis, both models were additionally re-estimated controlling for position (indicator variables). Mixed-effects models with player random intercepts were considered; however, in this small sample they resulted in singular/unstable fits. Therefore, OLS with player-clustered standard errors was retained as the primary specification.

Residual-versus-fitted plots were inspected to evaluate functional form and heteroskedasticity, and Q–Q plots were used to assess departures from normality of residuals. Standardized residuals, leverage, and Cook's distance were examined to screen for outliers and influential observations. A small number of observations exceeded conventional influence thresholds (Cook's D > 4/n); however, re-estimating the models after excluding these cases did not change the direction of the key coefficients and only slightly altered their magnitude. Therefore, results are reported from the full sample. Because inference relies on player-clustered standard errors, diagnostics were primarily used to identify influential cases rather than to enforce strict normality. To address the anti-conservativeness of cluster-robust standard errors under few clusters, the three Holm-significant associations were re-examined using a wild cluster restricted bootstrap (Cameron-Gelbach-Miller) with the null imposed, Rademacher weights, and resampling at the player-cluster level (9,999 replications; 95% confidence intervals obtained by test inversion) (39). To assess the robustness of the ratio-scaled bench-press predictor, the BP/BM–xGF%Rel association was additionally re-estimated (i) with absolute bench-press 1RM and body mass entered as separate covariates, and (ii) using an allometrically scaled predictor (BP1RM·BM^−b^, with b estimated from a log–log regression of 1RM on body mass) (40).

## 3 Results

Across the raw, pre-deletion dataset (*n* = 54 player-seasons), both outcomes were centered near zero (xGF%Rel: M = −0.002, SD = 0.057; FF%Rel: M = −0.001, SD = 0.038). After *a priori* listwise deletion of the four player-seasons with missing predictor data, the analytic sample comprised *N* = 50 observations from 28 unique players (Tables 2, 3). Unless otherwise stated, all descriptive statistics and models refer to this analytic sample. The four excluded player-seasons had higher mean outcome shares than the retained sample (xGF%Rel: M = 0.044, SD = 0.082; FF%Rel: M = 0.044, SD = 0.026), whereas age, position, and season distributions were broadly comparable (Table 3). This difference was driven largely by two player-seasons from a single player; the remaining two excluded player-seasons had xGF%Rel values below the retained-sample mean. Missingness was confined to the strength, jump, and sprint tests: single-leg strength was missing in three of the four player-seasons, countermovement jump, 30 m sprint, and bench-press strength in two each, and estimated VO₂max in one, while age and body fat percentage were available for all four. All models included season fixed effects, and standard errors were clustered by player. Variance inflation factors (VIFs) were low overall (range: 1.32–2.08), indicating no problematic multicollinearity (VIFs: 30 m = 1.37; CMJ = 1.32; SLS/BM = 1.74; BP/BM = 2.08; body fat percentage = 1.47; age = 1.58; Estimated VO₂max = 1.90).

**Table 3:** Missingness check (retained, complete-case vs excluded).

Variable	Retained (*N* = 50)	Excluded (*N* = 4)
Age [years], M (SD)	28.44 (4.46)	29.25 (2.63)
Forwards, *n* (%)	31 (62.0)	3 (75.0)
Defensemen, *n* (%)	19 (38.0)	1 (25.0)
Season (22/23, 23/24, 24/25)	15, 18, 17	0, 2, 2
xGF%Rel, M (SD)	−0.0061 (0.0541)	0.0440 (0.0822)
FF%Rel, M (SD)	−0.0045 (0.0371)	0.0440 (0.0257)
Body fat [%], M (SD)	9.99 (2.77)	11.18 (0.80)
CMJ [cm], M (SD)/values	48.69 (6.22)	46.83, 44.00 (*n* = 2)
30 m [s], M (SD)/values	4.27 (0.14)	4.49, 4.13 (*n* = 2)
SLS/BM [rel], M (SD)/values	1.52 (0.20)	1.79 (*n* = 1)
BP/BM [rel], M (SD)/values	1.29 (0.15)	1.18, 1.21 (*n* = 2)
Est. VO₂max [mL·kg⁻^1^·min⁻^1^], M (SD)/values	57.45 (6.47)	48.0, 59.1, 55.3 (*n* = 3)

Both outcomes (xGF%Rel & FF%Rel) were observed for all four excluded player-seasons; exclusions arose solely from incomplete off-ice testing (listwise deletion). For predictors with missing data in the excluded group, individual available values are listed instead of M (SD) because the small number of player-seasons precludes meaningful dispersion estimates; retained-group values are the complete-case means (M and SD) reported in Table 2. Missing predictors among the excluded player-seasons were: SLS/BM (three), CMJ and 30 m (two each), BP/BM (two), and estimated VO₂max (one). BM, body mass; BP/BM, bench press/body mass; CMJ, countermovement jump; FF%Rel, relative Fenwick for percentage; M, mean; N, number of player-seasons; n, number of player-seasons within each positional category (Forwards, Defensemen); SD, standard deviation; SLS/BM, single-leg squat/body mass; VO₂max, maximal oxygen uptake; xGF%Rel, relative expected goals for percentage.

For clarity, findings are presented in order of their statistical support under multiplicity control. Three associations survived Holm correction and are reported first — a positive BP/BM–xGF%Rel association, a positive CMJ–FF%Rel association, and a negative estimated-VO₂max–FF%Rel association — followed by predictors that reached significance only at the uncorrected level and those that did not. For xGF%Rel, the season-adjusted OLS model explained a modest share of variance (R^2^ = 0.312; adjusted R^2^ = 0.157; Table 4). Among the seven prespecified, z-standardized predictors, relative bench-press strength (BP/BM) showed a clear positive association (*β* = .021, 95% CI 0.009–0.033, *p* = .002). Estimated VO₂max (*β* = −.022, *p* = .026) and CMJ (*β* = .018, *p* = .039) were also significant at the uncorrected level, whereas age (*p* = .169), 30 m (*p* = .385), body fat percentage (*p* = .710), and SLS/BM (*p* = .772) were not. After Holm correction across the seven predictors, only BP/BM remained significant for xGF%Rel (p_Holm = .011; Figures 1,2). With BP/BM z-standardized, a 1-SD increase was associated with ≈ + 2.1 percentage points higher xGF%Rel.

**Table 4:** OLS regression models predicting xGF%Rel and FF%Rel.

Predictor (z-standardized)	xGF%Rel (5-on-5)	FF%Rel (5-on-5)
30 m	−0.009 (0.0102) [−0.030, 0.012] 0.385 1.0000	−0.012 (0.0050) [−0.023, −0.002] 0.021 0.1063
CMJ	0.018 (0.0084) [0.001, 0.036] 0.039 0.1939	0.018 (0.0051) [0.007, 0.028] 0.002 0.0118
SLS/BM	0.003 (0.0110) [−0.019, 0.026] 0.772 1.0000	0.007 (0.0061) [−0.006, 0.019] 0.285 0.8550
BP/BM	0.021 (0.0060) [0.009, 0.033] 0.002 0.0106	0.010 (0.0050) [−0.001, 0.020] 0.065 0.2610
Estimated VO₂max	−0.022 (0.0095) [−0.042, −0.003] 0.026 0.1564	−0.019 (0.0062) [−0.031, −0.006] 0.006 0.0352
Body fat %	0.003 (0.0089) [−0.015, 0.022] 0.710 1.0000	0.004 (0.0063) [−0.009, 0.017] 0.500 0.9995
Age	0.008 (0.0060) [−0.004, 0.021] 0.169 0.6764	0.001 (0.0044) [−0.008, 0.010] 0.838 0.9995
Observations	50	50
Players (clusters)	28	28
R-squared	.312	.358
Adj. R-squared	.157	.214

Cells report standardized coefficients (β) with player-clustered robust standard errors in parentheses; 95% CI in square brackets on the second line; the uncorrected p-value on the third line; and the Holm-adjusted p-value on the fourth line. All models include season fixed effects. Inference uses t distribution with df = number of player clusters - 1. 30 m was inverted so that higher values reflect faster performance. 5-on-5, five-on-five even-strength play; 95% CI, unadjusted 95% confidence interval; BP/BM, bench press/body mass; CMJ, countermovement jump; est. VO₂max, estimated maximal oxygen uptake; FF%Rel, relative Fenwick for percentage; p_Holm, Holm-adjusted p-value across the seven predictors within each outcome; SE, cluster-robust standard error; SLS/BM, single-leg squat/body mass; xGF%Rel, relative expected goals for percentage, β, standardized regression coefficient;

**Figure 1:**
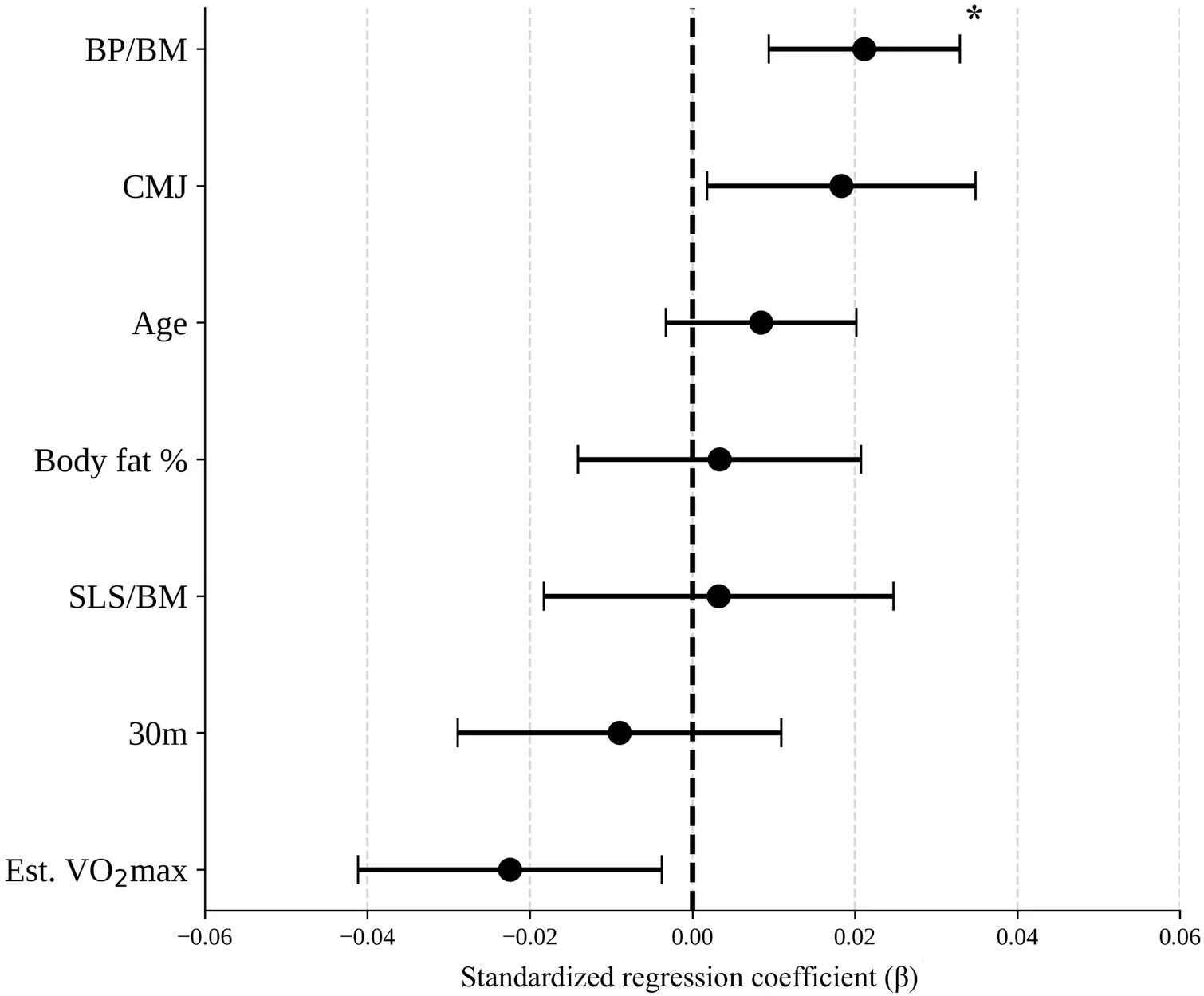
Standardized regression coefficients predicting xGF%Rel after holm correction. Points indicate standardized coefficients (*β*); horizontal bars show 95% confidence intervals. Predictors were z-standardized; 30 m speed was inverted so that higher values reflect faster performance. Models include season fixed effects; standard errors are clustered by player; *p*-values are Holm-corrected across the seven predictors. The asterisk (*) denotes p_Holm < 0.05. BP/BM, bench press/body mass; CMJ, countermovement jump; Est., estimated; SLS/BM, single-leg squat/body mass; xGF%Rel, relative expected goals for percentage.

**Figure 2:**
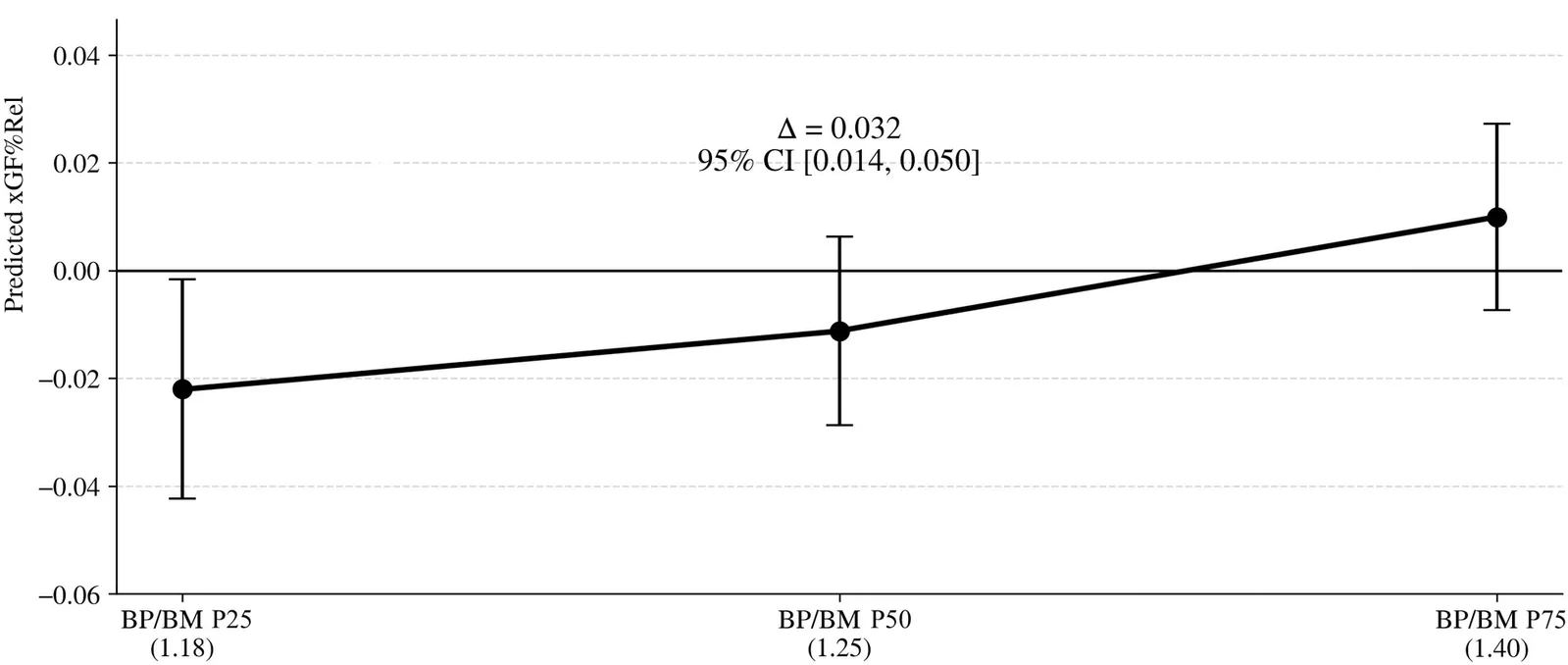
Predicted xGF%Rel at selected relative bench-press strength percentiles (P25, P50, P75). Points show model-predicted xGF%Rel for the 25th (P25), 50th (P50) and 75th (P75) percentiles of BP/BM; vertical bars indicate 95% confidence intervals. *Δ* denotes the predicted difference between P75 and P25 (P75 - P25); values in parentheses are the BP/BM ratio levels for each percentile, respectively. BP/BM, bench press/body mass; xGF%Rel, relative expected goals for percentage.

For FF%Rel, model fit was modest (R^2^ = 0.358; adjusted R^2^ = 0.214; Table 4). CMJ was positively associated (*β* = .018, 95% CI 0.007–0.028, *p* = .002) and estimated VO₂max negatively (*β* = −.019, 95% CI −0.031 to −0.006, *p* = .006). 30 m was negative at the uncorrected level (*β* = −.012, *p* = .021), while BP/BM (*p* = .065), SLS/BM (*p* = .285), body fat percentage (*p* = .500), and age (*p* = .838) were not significant. After Holm correction, CMJ (p_Holm = .012; Figure 3) and estimated VO₂max (p_Holm = .035) remained significant for FF%Rel (Figure 4). With CMJ z-standardized, a 1-SD increase was associated with ≈ + 1.8 percentage points higher FF%Rel.

**Figure 3:**
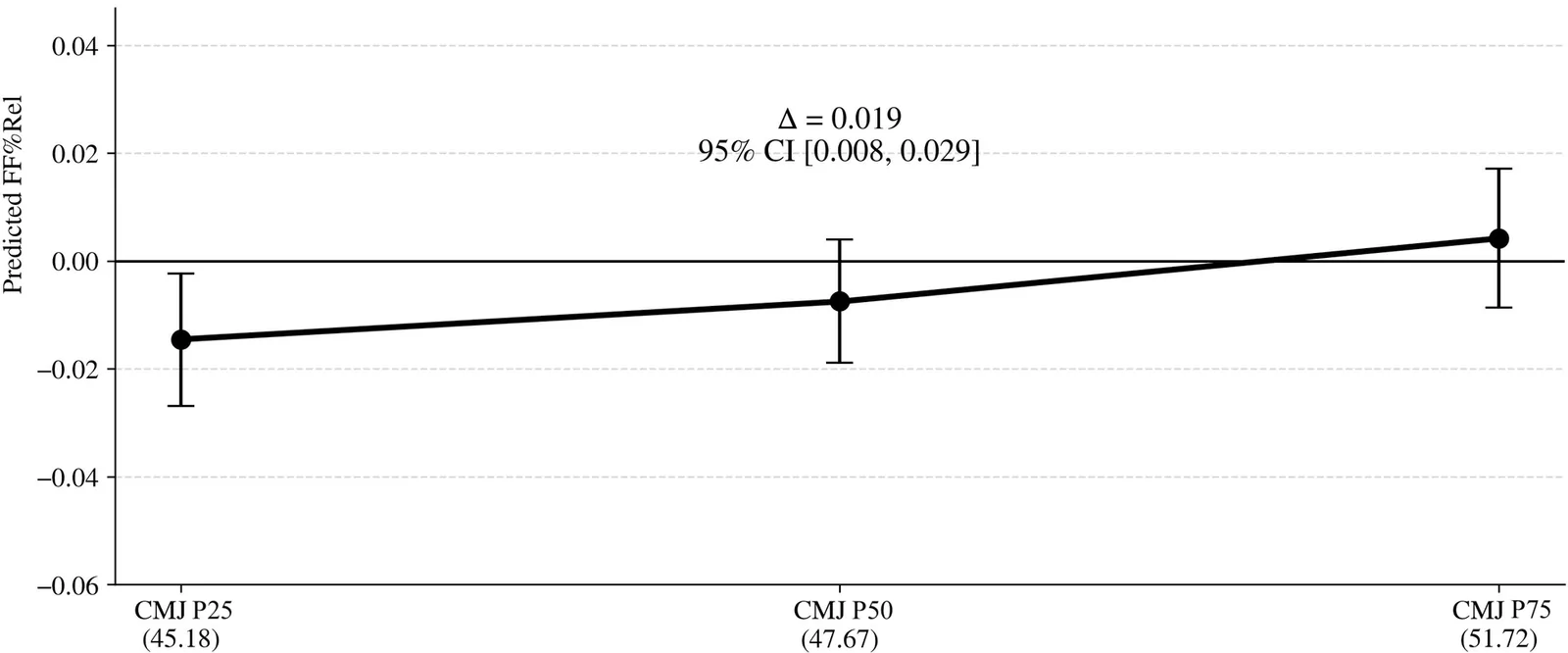
Predicted FF%Rel at selected countermovement jump percentiles (P25, P50, P75). Points show model-predicted FF%Rel for the 25th (P25), 50th (P50) and 75th (P75) percentiles of CMJ; vertical bars indicate 95% confidence intervals. *Δ* denotes the predicted difference between P75 and P25 (P75 - P25); values in parentheses are the CMJ levels (in cm) for each percentile. CMJ, countermovement jump; FF%Rel, relative Fenwick for percentage.

**Figure 4:**
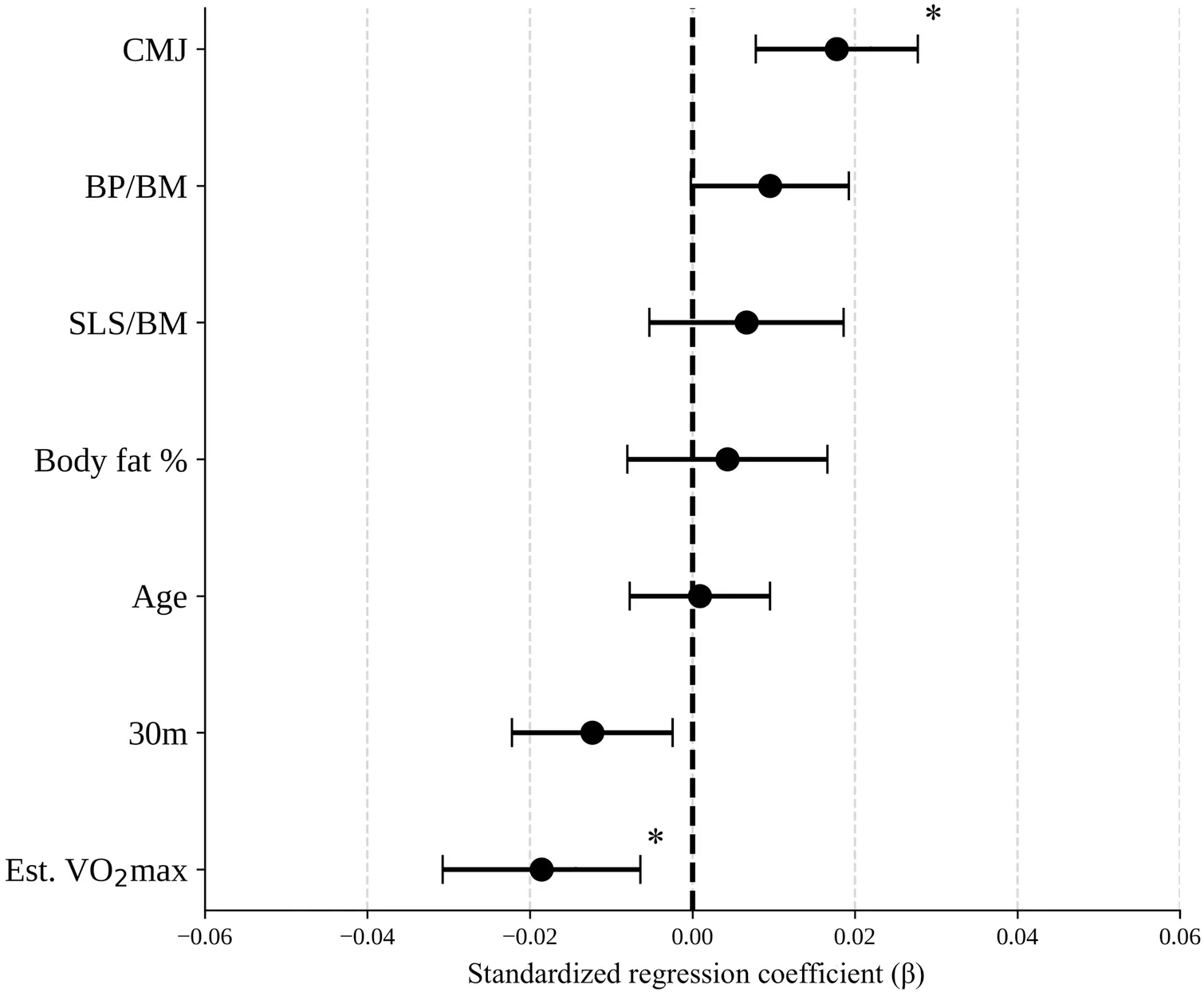
Standardized regression coefficients predicting FF%Rel after Holm correction. Points indicate standardized coefficients (*β*); horizontal bars show 95% confidence intervals. Predictors were z-standardized; 30 m speed was inverted so that higher values reflect faster performance. Models include season fixed effects; standard errors are clustered by player; *p*-values are Holm-corrected across the seven predictors. The asterisk (*) denotes p_Holm < 0.05. BP/BM, bench press/body mass; CMJ, countermovement jump; Est., estimated; FF%Rel, relative Fenwick for percentage; SLS/BM, single-leg squat/body mass.

Sensitivity analyses additionally adjusting for playing position produced the same substantive picture. For xGF%Rel, BP/BM remained positive and Holm-significant (p_Holm = .019; model fit R^2^ = 0.312; adjusted R^2^ = 0.135), while the other predictors did not survive Holm. For FF%Rel, CMJ (p_Holm = .013) and estimated VO₂max (p_Holm = .038) remained Holm-significant; overall fit was similar (R^2^ = 0.361; adjusted R^2^ = 0.198).

As a further sensitivity analysis addressing the dual role of the first author, who is both a participant in the dataset and the analyst, both models were re-estimated after excluding the author's own two player-seasons (*N* = 48 from 27 player clusters). The substantive pattern was largely preserved. BP/BM remained positively associated with xGF%Rel and Holm-significant (*β* = .020, 95% CI 0.007–0.033, p_Holm = .022), and CMJ remained positively associated with FF%Rel (*β* = .019, 95% CI 0.010–0.027, p_Holm = .0008); both were confirmed under wild cluster bootstrap inference (*p* = .012 and *p* = .011, respectively) and under position adjustment (p_Holm = .027 and.005). The negative estimated-VO₂max–FF%Rel association was directionally and numerically stable (*β* = −.018) and remained significant at the uncorrected level (*p* = .009); its Holm-adjusted value shifted marginally above threshold (p_Holm = .057), while the wild cluster bootstrap continued to support it (*p* = .014). Model fit was comparable to the full sample (xGF%Rel: R^2^ = 0.319, adjusted R^2^ = 0.158; FF%Rel: R^2^ = 0.383, adjusted R^2^ = 0.237), and all variance inflation factors remained ≤1.9.

As a targeted sensitivity analysis, each of the three coefficients that survived Holm correction was re-examined under the wild cluster restricted bootstrap. These bootstrap *p*-values are unadjusted for multiplicity and pertain only to the three selected coefficients; they therefore do not independently reproduce the Holm-controlled inference. All three remained significant at the unadjusted level: BP/BM–xGF%Rel (*p* = .011; 95% CI 0.008–0.037), CMJ–FF%Rel (*p* = .021; 95% CI 0.002–0.028), and estimated VO₂max–FF%Rel (*p* = .008; 95% CI −0.034 to −0.005). For the estimated-VO₂max–FF%Rel association, the like-for-like comparison of unadjusted inference — cluster-robust *p* = .006 versus bootstrap *p* = .008 — indicates a slight increase in the *p*-value under bootstrap resampling, while remaining below.05. For the ratio-scaled bench-press predictor, the positive BP/BM–xGF%Rel association was directionally consistent across re-specifications, but statistical support was not independent of body-mass specification. With absolute bench-press 1RM and body mass entered separately, 1RM remained positively associated with xGF%Rel and marginally survived Holm correction (*β* = .022, *p* = .006, p_Holm = .047), with body mass carrying an independent negative coefficient (*β* = −.031, *p* = .028) and all variance inflation factors ≤2.9. The allometric exponent was b = 0.54 (95% CI 0.20 to 0.88), estimated by ordinary least squares log–log regression of bench-press 1RM on body mass; because this estimate did not account for repeated player-seasons, the standard error was additionally clustered by player, which widened the interval substantially (95% CI −0.08 to 1.15) and indicates that the scaling exponent was imprecisely estimated in this sample. Under allometric scaling, the association remained positive and significant at the uncorrected level (*β* = .016, *p* = .026) but did not survive Holm correction (p_Holm = .19).

## 4 Discussion

Across three DEL seasons (*N* = 50 player-seasons from one club), only a small subset of preseason off-ice measures showed Holm-significant associations with tracking-derived, 5-on-5 performance shares. For xGF%Rel, the central result was a positive association with relative bench-press strength (BP/BM) that remained significant after Holm correction. In contrast, for FF%Rel, the predictors that survived Holm correction in the primary model were countermovement jump performance (CMJ; positive) and estimated VO₂max (negative), while BP/BM did not. Overall model fit was modest-to-moderate, which aligns with the expectation that in-game impact is shaped by multiple factors beyond generic off-ice fitness (2, 14, 15, 41). Because the design is observational and predictors precede outcomes only in measurement time rather than experimentally, none of the reported associations should be read as evidence of a training-induced effect.

The central finding for xGF%Rel was the positive association with BP/BM, which was Holm-significant in the primary model and preserved in direction and approximate magnitude under position adjustment and author exclusion. This support was, however, specification-dependent: the association did not survive Holm correction under allometric scaling (p_Holm = .19). Its direction is nonetheless mechanistically plausible: greater relative upper-body strength may support repeated contact and puck-control actions that are more likely to influence chance quality than shot quantity, including puck protection under pressure, net-front and board-battle positioning, opponent separation from the puck, and upper-body stabilization during passing/shooting under contact (15, 42). These micro-actions may cumulatively translate into longer offensive-zone possessions and improved access to dangerous shooting locations, consistent with the construct captured by expected-goals frameworks, which emphasize shot-quality-weighted process rather than attempt counts (16, 19). At the same time, this result should be interpreted strictly as a between-player association at the player-season level; it does not establish that increasing BP/BM within an individual will produce a proportional increase in xGF%Rel. This mechanistic reading requires an important qualification. When bench-press strength and body mass were entered separately, the strength term remained positive (*β* = .022) but body mass carried an independent negative coefficient (*β* = −.031), and under allometric scaling the association no longer survived Holm correction. These re-specifications preserve the positive direction but do not establish that the ratio result is independent of how body mass enters the model. The present data therefore cannot distinguish an absolute-strength/contact pathway — the mechanism outlined above — from alternatives that would produce the same ratio pattern, including a strength-to-mass relationship, mobility or skating-economy advantages associated with lower mass, deployment and role differences correlated with body size, or model-specification effects arising from ratio scaling. The negative body-mass coefficient should not be read simply as a “lighter-player advantage,” because it is model-specific, is reported without multiplicity adjustment, and is estimated imprecisely alongside a poorly determined scaling exponent (b = 0.54; player-clustered 95% CI −0.08 to 1.15). The direction of the body-mass term is, moreover, not consistent across the small existing literature: Peyer and colleagues reported a positive association between body mass and plus/minus among defensemen (42), underscoring that the present negative coefficient should not be generalized. Accordingly, the contact and puck-protection mechanisms are offered as one plausible interpretation among several that the current design cannot adjudicate. This cautious interpretation is consistent with broader evidence showing that off-ice physical tests translate only selectively to in-game performance metrics in ice hockey (7, 8, 14). The pattern that BP/BM was Holm-significant for xGF%Rel but not for FF%Rel is also coherent. A strength-related effect may be more visible in the conversion of possessions into higher-quality chances than in the raw volume of unblocked attempts, while FF%Rel is often more sensitive to contextual factors such as role, deployment, and team tactical environment (31, 32, 43).

For FF%Rel, CMJ showed a robust positive association after multiplicity correction. Lower-limb explosiveness relates to jump-based skating-speed qualities in ice hockey players (11); the CMJ–FF%Rel link was interpreted as a hypothesized pathway via short-burst skating and attempt generation, which (11) did not directly test. Recent evidence relating CMJ-derived neuromuscular qualities to skating performance is consistent with this hypothesized pathway; the most directly related study, however, examined jump kinetics and plantar forces rather than contact-mat jump height, assessed sprint-skating rather than in-game outcomes, and used a small national-level sample (44). Transfer from off-ice tests to on-ice performance more generally appears context-dependent rather than uniform (13).

Taken together with the xGF%Rel results, the split remains internally consistent: CMJ aligns more with creating/sustaining attempt volume (FF%Rel), whereas BP/BM aligns more with chance quality (xGF%Rel).

The negative association after Holm adjustment between estimated VO₂max and FF%Rel (*β* = −.019; ≈ −1.9 percentage points in FF%Rel per 1-SD increase) should not be interpreted as evidence that aerobic capacity is detrimental (2, 4). Rather, it likely reflects role-mediated confounding: players with higher aerobic capacity may be deployed in defensive shutdown roles or against the opponent's top lines, which can suppress territorial share metrics like FF%Rel. These ‘defensive specialists’ may prioritize gap control and shot-blocking over territorial dominance, leading to a statistical inversion where higher fitness appears associated with lower relative shot shares. This interpretation is consistent with prior professional-hockey findings that generic off-ice fitness tests show only trivial-to-small associations with match-performance outcomes, with age rather than fitness emerging as the dominant correlate (15). Future research should integrate Quality of Competition (QoC) and Quality of Teammates (QoT) — summary indices of, respectively, the average calibre of the opponents a player faces and of his own linemates while on the ice, commonly derived from their ice time or performance — to help separate a player's physiological capacity from the tactical and contextual circumstances of his deployment. In addition, the cycling-based estimate of aerobic capacity is a proxy — obtained from a proprietary algorithm whose validity for this specific cycle-ergometer protocol has not been established — that may not capture skating-specific endurance demands (45–47), adding uncertainty that future work should address with sport-specific or on-ice assessments.

Other predictors showed weak or non-robust associations. Off-ice 30 m sprint performance did not survive Holm correction, which is unsurprising given that straight-line running speed is an incomplete proxy for skating-specific acceleration mechanics, edge work, and multi-planar/multi-directional actions that shape 5-on-5 possession and chance generation (25, 48). Single-leg strength scaled to body mass (SLS/BM) and body fat percentage did not meaningfully explain either outcome under corrected inference, and given ratio scaling and the limited sample size, coefficients on these terms are particularly vulnerable to interpretability issues. Age also showed no meaningful association with either xGF%Rel or FF%Rel in this cohort, which is plausible in a relatively narrow professional age range and for team-relative share metrics that are less directly driven by seniority-linked opportunity than box-score scoring outputs (15). Sensitivity analyses adjusting for position did not change the substantive picture, suggesting that the key patterns are not simply a by-product of forward/defense composition.

Accordingly, the percentile-based predictions (Figures 2,3) should be read as descriptive, sample-specific illustrations of effect direction and magnitude rather than validated thresholds, as they may not generalize beyond this team, season context, and vendor model.

These metric-specific associations are consistent with a broader invasion-sport literature in which physical testing transfers to in-game output only inconsistently and in a context-dependent manner. In rugby, unadjusted performance indicators can distort the relationships they aim to capture (49), positional and contact demands diverge once expressed relative to playing time (50), heterogeneous basketball test batteries have prompted calls for position-specific reporting (51) and in soccer and handball fitness relates to match demands only selectively and by position, level, or sex (52, 53). Taken together, this reinforces the interpretation of the present findings as capacity-specific and context-mediated rather than reflecting a global fitness–performance relationship.

Overall, the findings provide only partial support for the broad hypothesis that superior off-ice fitness translates into superior in-game impact. Rather than a general relationship, the observed associations are capacity-specific. Upper-body relative strength was positively associated with expected-goals impact and lower-limb explosiveness was the most consistently supported correlate of unblocked shot share, remaining Holm-significant across every sensitivity specification in which it was re-estimated. The aerobic proxy showed a negative association with Fenwick share that was Holm-significant in the primary model but fell just above the adjusted threshold after exclusion of the first author's player-seasons (p_Holm = .057); this association is, in any case, plausibly context-mediated rather than a detrimental effect of aerobic capacity *per se*.

### 4.1 Limitations

The interpretability of these findings is constrained by several limitations.

First, the dataset is small and drawn from a single team (*N* = 50 player-seasons; 28 skaters; no goaltenders), which limits generalizability to other teams, leagues, and to women's hockey (8). In addition, the inclusion criterion of ≥50% games played and listwise deletion may introduce survivorship/availability bias by under-representing injured players, healthy scratches, or short-tenure roster members.

Second, although xGF%Rel and FF%Rel are team-relative share metrics, they remain sensitive to contextual and deployment-related factors, including zone starts, score effects, QoT & QoC, and matchup structure (31, 32, 43). This sensitivity also limits the extent to which on-ice performance can be inferred from generic off-ice tests, because commonly used 5-on-5 advanced metrics are shaped by numerous endogenous and exogenous determinants that such tests cannot adequately capture in the situational and interactive context of ice hockey (7, 19). Relevant determinants include tactical deployment and usage, players’ game intelligence and tactical awareness, sport-specific technical skills (e.g., skating, passing, stickhandling, and shooting), and psychological characteristics such as mental toughness and related attributes. In addition, coaching systems and utilization patterns may vary across seasons; in the present sample, the team was led by two head coaches, with a coaching change after the 2022/23 season. Beyond season fixed effects, these influences were not explicitly modeled and may therefore confound the observed associations between fitness and performance. Relatedly, player-level 5-on-5 TOI was not available, so player-seasons entered the models unweighted and the distribution of 5-on-5 exposure across the sample could not be characterised. Equal weighting gives player-seasons with limited 5-on-5 min the same influence as those with heavy usage, which adds noise to the on-off relative shares and is most consequential for the outcome most exposed to deployment. This caveat applies with particular force to the negative estimated-VO₂max–FF%Rel association: because its most plausible reading is role- and usage-mediated, and because we could neither TOI-weight nor impose a minimum 5-on-5 exposure threshold, a genuine physiological signal cannot be fully separated from deployment-driven variation in territorial share.

Third, inference is fragile in small clustered samples. Players contribute repeated observations and standard errors are clustered at the player level; with only 28 clusters, p-values—especially those near.05—can be sensitive to finite-sample properties and should be interpreted alongside effect sizes (54).

Additional measurement and timing constraints apply. Predictors represent single preseason snapshots, whereas outcomes summarize months of play, creating temporal misalignment and potential regression dilution. Because several predictors are proxy measures (e.g., estimated VO₂max, estimated 1RM, skinfold-derived body composition), classical measurement error may attenuate regression coefficients toward zero. Therefore, the estimated associations should be interpreted conservatively, and true effects—if present—may be larger in magnitude than observed. Because in-sample reliability was not quantified, effects sitting close to the Holm-adjusted significance threshold — most notably the estimated-VO₂max association with FF%Rel (p_Holm = .035) — should be interpreted with particular caution, as unquantified measurement error may inflate or deflate near-threshold coefficients. Skinfold assessments are susceptible to technique-related effects, hydration status, and inter-rater variability; however, familiarization and test-retest reliability were not quantified. Likewise, contact-mat-derived jump height, cycle-ergometer outputs, and sprint timing-gate measurements may be affected by instrument-related technical error. For movements allowing individualized stance or grip, participants used a self-selected setup to reflect applied practice. This increases ecological validity but may introduce additional between-session variability if the self-selected setup cannot be replicated precisely across testing sessions/seasons. Moreover, ratio-scaled predictors (…/BM) can introduce mathematical coupling and suppressor effects even when conventional multicollinearity diagnostics (e.g., VIFs) appear low (39, 55, 56); however, sensitivity analyses decoupling bench-press strength from body mass preserved the direction of the BP/BM–xGF%Rel association but did not establish its independence from body-mass specification: statistical support was strongest in the covariate specification and did not survive Holm correction under allometric scaling. As discussed above, the negative body-mass coefficient and the imprecisely estimated scaling exponent mean that an absolute-strength/contact pathway cannot be distinguished from strength-to-mass, mobility, deployment, or model-specification pathways.

Finally, vendor and reproducibility constraints should be noted. Both tracking event detection and the xG model are proprietary vendor algorithms whose internal logic and version histories could not be independently audited; their classification accuracy therefore cannot be verified here, and event misclassification cannot be excluded. No validation evidence was available for this proprietary event- and xG-classification layer. The accuracy of the underlying positional tracking is a separate matter: an indoor ice-hockey local positioning system has been validated for external-load, speed, and acceleration measurement (57); the available indoor-LPS validation evidence, however, pertains to a different commercial system (Kinexon) from the one underlying the present dataset (Wisehockey), and validation studies in team sports report that distance and speed measurement errors depend on the measured variable, environment, and filtering method, typically ∼1–8% (58, 59). These positional- and distance-measurement estimates should not be extrapolated to the proprietary event-detection or xG-classification algorithms, which those studies did not evaluate. Data-sharing restrictions also prevent full open replication (8).

### 4.2 Practical applications

The following suggestions should be interpreted as evidence-informed starting points for internal monitoring and hypothesis testing rather than causal training recommendations or universal decision rules; their utility should be evaluated locally and re-evaluated as more seasons and players accumulate. Two further caveats bound the scope of these suggestions. First, the present models estimate between-player associations from single preseason predictors and season-aggregated outcomes; they do not evaluate within-athlete change, training response, monitoring frequency, retesting strategies, or the reliability and decision value of short-window share metrics. Any recommendation concerning testing frequency, within-athlete monitoring, or short game windows is therefore an external, general-practice consideration that was neither tested nor supported directly by the present analyses. Second, given the modest model fit and the sensitivity of the BP/BM result to scaling specification, these suggestions are offered as hypotheses for local evaluation rather than as validated decision rules.

The findings support a deliberately lean, context-aware approach to preseason testing rather than expanding test batteries by default (41). Practically, measures that plausibly map onto the play-driving processes captured by share-based metrics should be prioritized, and additional tests should only be added when they provide clear decision value (14). Accordingly, a compact preseason core battery may be considered as a pragmatic starting point in similar operational contexts: BP/BM as an indicator of relative upper-body strength and CMJ as a measure of lower-body explosiveness assessed as best-of-three attempts, complemented by basic anthropometrics (body mass and stature), with body fat percentage used primarily for contextual profiling rather than as a performance KPI (41, 60). Off-ice sprint performance should not be treated as a stand-alone decision criterion for 5-on-5 impact, even if it remains relevant to general speed development (10, 48). When aerobic capacity is assessed, the applied purpose should be specified explicitly (e.g., readiness, recovery tolerance, availability), and measurement and interpretation should be aligned with that purpose (61). The percentile-based predictions (Figures 2,3) are best used to communicate directionality and plausible magnitude of associations to staff, not as validated thresholds. Beyond efficiency, streamlining the battery toward measures with demonstrable explanatory value—and redirecting monitoring resources toward athlete readiness, recovery tolerance, and availability—aligns testing practice with athlete health and well-being, contributing to Sustainable Development Goal 3 (Good Health and Well-being).

The two off-ice measures that carried explanatory value in the present between-player models — relative bench-press strength and countermovement jump — may be reasonable candidates to prioritize when a club chooses to monitor off-ice qualities in season. The present study does not, however, evaluate monitoring frequency, retesting protocols, or within-athlete responsiveness, and it provides no basis for a specific testing schedule. The present analyses do not evaluate the stability or decision value of xGF%Rel and FF%Rel over short game windows. Any in-season use of these metrics would require explicit adjustment for 5-on-5 exposure and deployment — neither of which was available here — because short on/off windows can be highly unstable without such adjustment. The reliability of short-window share metrics therefore remains to be established and should not be assumed (14, 32).

In terms of decision-making, a transfer-first logic may be adopted as a general principle — and not as a finding of this study, which does not model within-athlete change: off-ice gains are most plausibly meaningful when accompanied by movement in the hypothesized on-ice mechanisms and, subsequently, in the share-based metrics. Establishing whether such within-athlete transfer occurs requires longitudinal, within-player designs that were beyond the scope of the present between-player analysis (14, 48). If gym metrics improve without corresponding movement in on-ice indicators, the priority should be to revisit technical and tactical constraints and interpret results in light of deployment and role, rather than expanding the test battery (1, 7). Because the present models quantify between-player associations in a single-club sample, practical use should focus on within-team learning rather than generalizable benchmarks.

### 4.3 Conclusion

In this DEL cohort (*N* = 50 player-seasons from one club across three seasons), preseason off-ice fitness showed selective, capacity-specific associations with advanced 5-on-5 impact metrics rather than a broad “more fit = better” pattern. After multiplicity control in the primary models, BP/BM was the only Holm-significant correlate of xGF%Rel, and CMJ (positive) and estimated VO₂max (negative) were Holm-significant for FF%Rel. Across sensitivity specifications this support was not uniform: the CMJ–FF%Rel association remained Holm-significant throughout, whereas the BP/BM association did not survive allometric scaling and the estimated-VO₂max association fell just above the Holm threshold after author exclusion, even though both retained their direction and approximate magnitude. This pattern suggests that different physical capacities may map onto different on-ice processes (chance quality versus unblocked attempt share). However, the overall explanatory signal of generic off-ice testing remains limited in magnitude.

Methodologically, these results are associative and reflect a small, single-team dataset. Decision-grade inference requires larger multi-team longitudinal data with explicit deployment adjustment (e.g., 5-on-5 TOI, zone starts, QoT/QoC), reproducible model scaffolding, and routine reporting of measurement reliability for both fitness tests and tracking-derived KPIs.

Practically, the most meaningful takeaway is not to chase single test numbers, but to evaluate whether changes in trainable capacities translate into the relevant, proximal on-ice behaviors and—only then—into improved 5-on-5 share-based metrics. At the same time, the findings highlight a key limitation: 5-on-5 advanced metrics are influenced by many factors that generic off-ice tests cannot capture in the situational, interactive context of ice hockey, including deployment/usage, game intelligence, sport-specific technical skills, tactical skills/level, and psychological characteristics.

## Data Availability

The datasets presented in this article are not readily available because they contain sensitive personal performance data of professional athletes and are subject to data protection agreements with the participating club and the league's official tracking provider. Data were analyzed under restricted access, and results are reported in aggregated form to safeguard the identity of individual players, with the exception of the individual available values for the four excluded player-seasons in Table 3, reported because dispersion estimates would be uninformative for so few rows. Requests to access the datasets should be directed to PS, spornbergerpeter@gmail.com; access may be granted only within the limits of the underlying data protection agreements.
