# Supplementary material for: Relative bench-press strength and jump power are associated with distinct tracking-based in-game shares in professional ice hockey
Source: Front Sports Act Living. 2026 Oct 13;8:1917193. doi: 10.3389/fspor.2026.1917193 (PMC13710354; doi:10.3389/fspor.2026.1917193)
Supplement: Supplementary file 2 [file Supplementaryfile1.pdf]

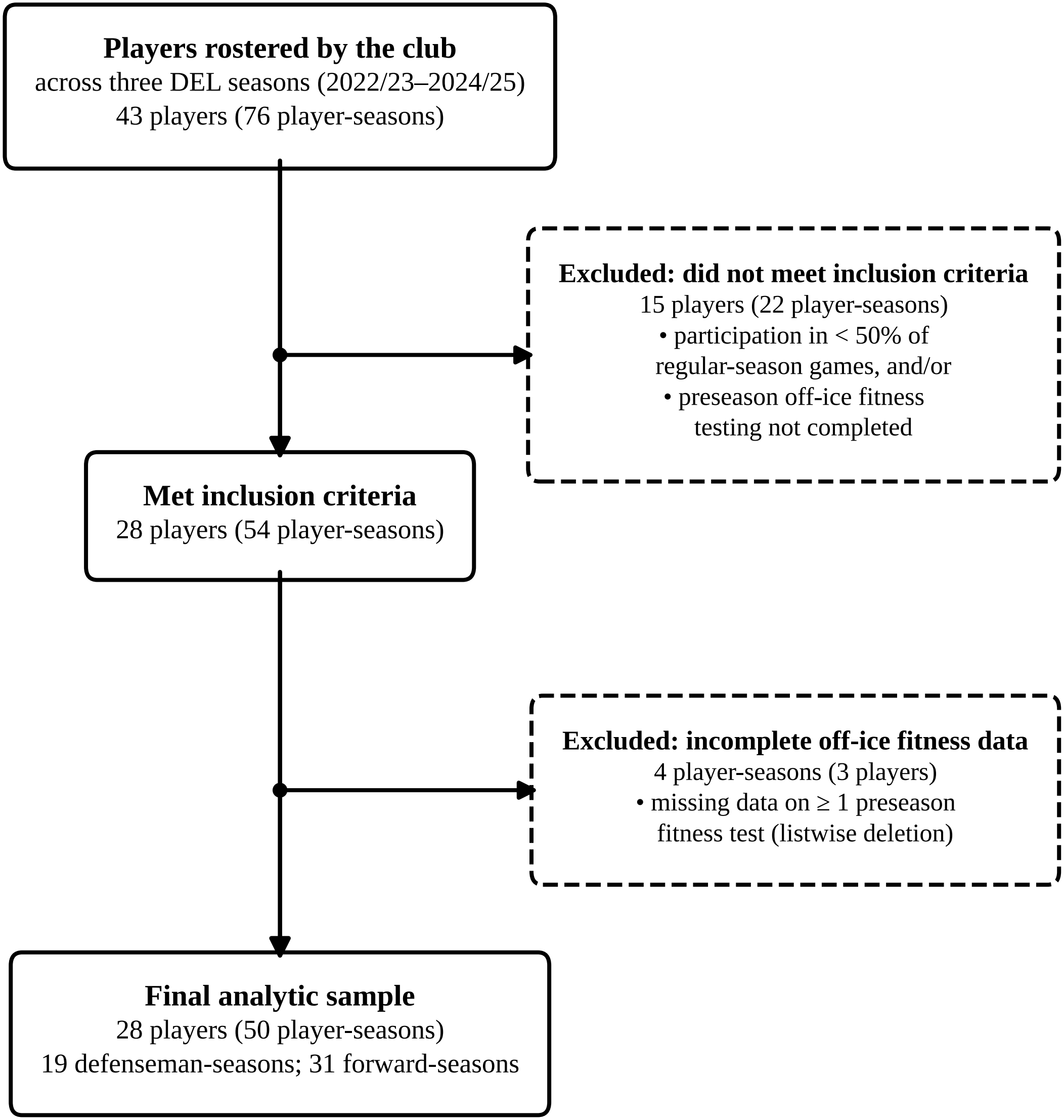
Players rostered by the club
across three DEL seasons (2022/23–2024/25)
43 players (76 player-seasons)
Excluded: did not meet inclusion criteria
15 players (22 player-seasons)
• participation in < 50% of regular-season games, and/or
• preseason off-ice fitness testing not completed
Met inclusion criteria
28 players (54 player-seasons)
Excluded: incomplete off-ice fitness data
4 player-seasons (3 players)
• missing data on ≥ 1 preseason fitness test (listwise deletion)
Final analytic sample
28 players (50 player-seasons)
19 defenseman-seasons; 31 forward-seasons
